# The Week's Work

**Published:** 1911-06-03

**Authors:** 


### The Week's Work.

### THE BRITISH HOSPITALS ASSOCIATION MEETING IN LONDON.

By the time this issue is in the hands of our readers the special Conference on the Insurance Bill called by the British Hospitals Association will have begun. At three o'clock on Friday the delegates met at St. George's Hospital, and, as we go to press, we are given to understand that the Conference will probably begin with a paper by Mr. Charles Lupton, the well-known Treasurer of Leeds General Infirmary, on the attitude towards the Government's Insurance Bill which the voluntary hospitals ought to adopt. We congratulate the British Hospitals Association on the promptitude with which it has taken up this matter, as we urged it to do the moment the Bill was brought out, and we are confident that its Conference will generate a policy on which the voluntary hospitals will be glad to unite. The formation of such a policy has been assisted and the work of the Conference already begun in these columns, which have produced a crop of opinions that has attracted wide attention, with the result of a preliminary clearing of the ground. Our correspondence columns this week produce evidence of the keenness with which the views of the hospital secretaries are being followed, and we hope next week to give an authoritative report of the British Hospitals Association's Conference, which will then set the seal of authority on the discussion and produce the accepted leadership which could not be forthcoming until the Conference had met.

### TORQUAY CASE TO BE RETRIED.

The result of the appeal of the Torquay Corporation from the decision of the County Court, which awarded damages for the alleged negligence of its servants at the borough isolation hospital, under circumstances detailed in The Hospital of May 6, will give rise to much interest. Mr. Justice Pickford, in the Divisional Court of the King's Bench Division, last week delivered reserved judgment to the effect that the case was remitted for a new trial, with leave to appeal. Mr. Pickford stated that the point to be considered was whether the Judge of the County Court was wrong in deciding that the Torquay Corporation, not having pleaded the Public Authorities Protection Act, 1893, could not avail themselves of its protection. Borough hospitals throughout the country will be affected most nearly by the ultimate decision, and the progress of the fresh trial will be watched with the keenest attention.

### "HOSPITALS AND CHARITIES" IN CORONATION YEAR.

The preface to this present year's edition of this book contains its own review in the following sentence: "The volumes of this book already published constitute a fairly complete history of hospital progress, and with the volumes of The Hospital newspaper and the four volumes of 'Hospitals and Asylums of the World' comprise as complete a library of reference on hospital subjects, with the plans of hospitals as they have been reconstructed, or erected, as is available." The long survey of the four historical volumes, the twenty-second yearly record which "Burdett's Hospitals and Charities" has become, and the weekly record for twenty-five years which these pages continue: here at least have been laid down architectonic sources for the flow of information. The Annual this year has the useful addition of the names of "the original and present architects of important hospitals." The chapter relating to the cost of in- and out-patients has been omitted for stated reasons, depending upon the general adoption of the uniform system of accounts. The index has been still further extended by the addition of the shorter and more colloquial names that many hospitals bear, and is now, in a vigorous motoring phrase, absolutely fool-proof. Nursing departments have been dealt with exhaustively, and that no want of modernity may be hinted, a searching paragraph of the preface is devoted to the recent Commission on the Poor Law, suggesting that general scheme of co-operation between it, the profession, and the hospitals which this journal has often advocated in the past few months. This summary is enough to prove that "Burdett's Hospitals and Charities" is absolutely indispensable to every modern hospital, and it would now be hard to find an institution in the Empire that is not included in it.

### A RECORD OF PROGRESS AT BOLTON.

We understand that a movement is now on foot to gain Royal recognition for the Bolton Infirmary. We hope this may be done, so that the recent progress which has been inaugurated at this institution may receive official recognition. The steps in this progress are worth recording. On February 12, 1910, we published a report on the Infirmary, and exactly a month later (page 700) were able to publish its prompt effect in the Board's decision to request the British Medical Association to nominate an expert to investigate the whole position of affairs. On July 16 we gave the full text of the Report of the gentleman appointed, who proved to be Dr. Mackintosh, of Glasgow. A comparison of his and Sir Henry's Reports makes most interesting reading, and the upshot was a series of largely similar recommendations, prominent among which was a plea for a new Nurses' Home. On September 17 (page 747) we hinted the probability of the extension of the hospital being undertaken as Bolton's memorial to King Edward. This has been done, and £25,000 has been raised, by means of which, we are informed, the new Nurses' Home and various structural alterations will be constructed. If these endeavours are capably presented in the petition which, we presume, the hospital in the usual course will present to the Home Secretary, we think that there can be little doubt that a favourable reply will not long be delayed. We believe that the hospital receives strong local support, the annual Hospital Saturday collections now, we understand, having reached £4,500, and freedom from debt is, of course, one of the strongest facts in the favour of any hospital petitioning to be styled "Royal." We went thoroughly into this general question in our issue of October 15 last, and can only hope that the Bolton Infirmary will be successful.

### THE NEW HOSPITAL DIRECTORY FOR THE U.S.A. AND CANADA.

We are glad to have received the first issue of Sutton's "Hospital and Institutional Directory," which is endeavouring to do for America what Burdett's "Hospitals and Charities" does for the Empire. The first issue of "Sutton" naturally has been a difficult achievement, but it is to be hoped that American hospitals will realise its value, so that the second and future volumes may include additional information to the name, address, and size of the institutions, which are all that is obtainable at present. Hospitals need educating in their responsibility as public institutions to raise their efficiency and increase their usefulness by combining to make their annual directories a success. It has been done here in the case of "Burdett," and will, we hope, be done in America, but it takes time. The compiler, by the way, is Mr. T. Sutton, the editor of the *International Hospital Record*.

### HOSPITALS AND TRACHOMATOUS PATIENTS.

The comparative rareness of trachoma in our out-patient departments does not make it less interesting to note that the Home Secretary is considering whether he might properly exercise his power of altering the definition of immigrant ship with the object of rendering the exclusion of trachomatous aliens still more effective. In replying to a question in the House of Commons, with reference to an increase in the ranks of trachomatous children admitted to the ophthalmic schools of the Metropolitan Asylums Board, Mr. Churchill said the number admitted in 1906 was 115; in 1907 the number fell to 82; but in 1910 it rose to 122. The largest number of the 122 cases, however, came from Southwark which, as the Home Secretary pointed out, is not one of the areas into which newly arrived aliens mostly go. Nevertheless, it is important that non-immigrant ships should be closely watched with the view to checking the importation of trachoma into the hospitals of this country.

### THE INSURANCE BILL'S EFFECT ON MEMORIAL SCHEMES.

A desire to perpetuate the memory of the late King has been the inspiration of so much hospital-construction during the last twelve months, and this has so often taken the form of a new or extended out-patient department, that the probable effects of the Government's Insurance Bill are being watched with the most anxious interest by the commemorating institutions. At Scarborough, for instance, an appeal has been issued for funds for an extended out-patient department as the town's memorial to King Edward. Some £5,000 has been appealed for, and over £3,000 has been raised. The additional new rooms proposed include a small theatre, a recovery room where doubtful casualty cases can be observed, new dental room and casualty room for both sexes. In short, a modern out-patient unit on the latest lines was in contemplation, indeed in progress. Now the secretary of the Scarborough Hospital, Mr. G. P. Adamson, is compelled to write "We shall, of course, have to take into consideration the Insurance Bill as to its probable effect on the scheme before proceeding." This is an example of the immediate effects of the Bill which has received no attention, but which gives more food for thought perhaps than its much-discussed ultimate possibilities.

### THIS WEEK'S DRUG MARKET.

A quiet tone pervades the drug market, and no improvement may be expected until the Whitsuntide holiday is well over. The Coronation holidays are also likely to interfere considerably with business. Few changes in value have taken place. Ergot of rye has again advanced in price, and the extreme figure of 5s. per lb. has already been paid; at the present time it might be difficult to buy even at this price. Cod-liver oil still shows a tendency to decline in value, partly as a result of the absence of demand during the hot weather which has prevailed of late. The price of opium is not only maintained, but there seems to be a likelihood of a further advance, if only small. Morphine and codeine are more likely to advance than to decline in value, in view of the market position of the raw material. Buchu leaves have again advanced in value. Balsam copaiba is tending rather dearer.

